# Change in depression during the COVID-19 pandemic among healthcare providers in Addis Ababa, Ethiopia

**DOI:** 10.7717/peerj.15053

**Published:** 2023-04-10

**Authors:** Firehiwot Workneh, Alemayehu Worku, Nega Assefa, Yemane Berhane

**Affiliations:** 1 Epidemiology and Biostatistics, Addis Continental Institute of Public Health, Addis Ababa, Ethiopia; 2 School of Public Health, College of Health Sciences, Addis Ababa University, Addis Ababa, Ethiopia; 3 College of Health and Medical Sciences, Haramaya University, Harar, Ethiopia

**Keywords:** COVID-19, Depression, Mental health, Healthcare providers, Addis Ababa, Ethiopia

## Abstract

**Background:**

The COVID-19 pandemic has increased mental health problems among healthcare workers globally. However, studies from low- and middle-income countries on this matter were minimal. This study assessed the change in depression prevalence during the first year of the COVID-19 pandemic and associated factors among healthcare providers in Addis Ababa, Ethiopia.

**Methods:**

We conducted surveys among healthcare workers in Addis Ababa at two-time points, September 2020 and October 2021. A total of 577 study participants were randomly selected based on registers obtained from professional associations. Computer-assisted telephone interviewing technique was used for data collection. The Patient Health Questionnaire (PHQ-9) was used to screen for depression. Multivariable logistic regression analysis was performed to identify potential factors associated with depression.

**Result:**

The prevalence of depression among healthcare workers was 2.3% (95% CI [1.1–4.8]) in Time 1 and 6.5% (95% CI [4.1–10.1]) in Time 2; nearly a three-fold increase in Time 2 compared to Time 1. The most frequently reported symptoms at both times based on the PHQ-9 item were having poor energy, sleep problem, and anhedonia, while reported suicidal ideation was less than 5%. Depression showed a positive and significant association with a positive COVID-19 test result (AOR 7.25 95% CI [1.32–39.4]) in Time 1, and with being a female healthcare provider (AOR 3.96 95% CI [1.08–14.51]) and lack of COVID-19 related policy or guidelines at the workplace (AOR 3.22 95% CI [1.11–9.35]) in Time 2.

**Conclusion:**

The prevalence of depression among healthcare workers tripled during the first year of the COVID-19 pandemic. Panic reaction to positive COVID-19 test result seems to have a negative effect at the beginning while lack of disease-specific prevention guidelines and comprehensive psychological interventions for healthcare providers had an adverse effect on the mental health of health workers.

## Introduction

Globally, the coronavirus disease (COVID-19) pandemic has contributed to an upsurge in mental health problems (Ettman et al., 2022; World Health Organization, 2022). The World Health Organization (WHO) estimated that in the first year of the COVID-19 pandemic, the prevalence of depression increased by 25% (United Nations, 2022), especially among vulnerable groups (Almeida et al., 2021). Previous studies have reported that healthcare professionals (HCPs) are highly susceptible to poor mental health outcomes during outbreaks as manifested by high levels of depression, stress, anxiety, and post-traumatic stress disorder (Abhery et al., 2021; Garcia et al., 2022). Increased demand for health services during outbreaks, accompanied by resource constraints in the health system, raises the risk of poor mental health among HCPs in low- and middle-income countries (LMICs) (Dawood, Tomita & Ramlall, 2022).

Despite the potential mental health impact of COVID-19, the health workforce protection was mostly centered on physical threats, particularly in LMICs (Dawood, Tomita & Ramlall, 2022). However, emerging and existing evidence indicates that the mental health impact of large-scale outbreaks has a more severe impact on the well-being of the health workforce (Kapetanos et al., 2021). Depression is one of the common mental health problems that healthcare providers face during outbreaks, which is characterized by a range of symptoms such as low mood, guilt, and worthlessness (Elliott et al., 2021; Pappa et al., 2020). This has been demonstrated by research that showed significant long-term psychological effects of infectious disease outbreaks such as Severe Acute Respiratory Syndrome (SARS) and Middle East Respiratory Syndrome (MERS) (Lee et al., 2018; McAlonan et al., 2007).

A study conducted in thirteen African countries reported a 30% increase in depressive symptoms among healthcare workers during the COVID-19 pandemic (Debes et al., 2021). A systematic review among HCPs in Africa indicated a wide range of depression prevalence which ranged from 12.5% to 71.9% (Olashore et al., 2021). Studies on mental health among HCPs during a specific phase of a pandemic showed heterogeneity in the prevalence of depression among HCPs, which might be related to the study’s setting, design and timing (Magnavita, Soave & Antonelli, 2021; Th’ng et al., 2021). Thus, indicating the need for longitudinal studies to accurately understand the extent and drivers of the problem (Magnavita, Soave & Antonelli, 2021). Different follow up studies conducted among healthcare workers found significantly worsened depression (Th’ng et al., 2021; Zhou et al., 2021). The succession of COVID-19 waves, which is made worse by the emergence and spread of new Severe Acute Respiratory Syndrome coronavirus *2* (SARS-CoV-2) variants with high transmission rates and virulence may have contributed to the rise in the prevalence rate of depression and other mental health outcomes (Zara et al., 2021). Poor mental health outcomes, specifically depression amongst HCPs, showed significant association with long working hours, female gender, nurse professions, younger age group, and comorbidity, which showed similarities among HCPs in both high and low and middle-income settings (Mulatu et al., 2021; Th’ng et al., 2021; Lai et al., 2020; Ayalew et al., 2021; Moitra et al., 2021; Chutiyami et al., 2022; Zara et al., 2021). Inadequate training and unavailability of guidelines at health facilities are among the occupational factors significantly associated with higher levels of depression(Bitew et al., 2022; Olashore et al., 2021).

To date, several cross-sectional studies have been conducted in Ethiopia to assess the prevalence of depression among HCPs. Nevertheless, there is limited published evidence on the mental health impacts of the COVID-19 pandemic among HCPs over the course of a year. This study aimed to evaluate the change in depression during the first year of the COVID-19 pandemic and determine factors associated with depression among healthcare providers.

## Materials & Methods

### Study design and setting

We conducted surveys among HCPs at two-time points (Time 1 and Time 2). The first survey was conducted in September 2020, and the second survey was done a year later in October 2021. The study was conducted in Addis Ababa, the capital city of Ethiopia with an estimated 5.2 million people (Central Statistical Agency, 2013). Addis Ababa had the highest burden of confirmed cases and death from COVID-19 in the country: 67% of the total cases and more than half of the COVID-19 attributed deaths (Abdella et al., 2021; EPHI, 2022). Addis Ababa has a better health infrastructure and the highest number of qualified medical personnel compared to other cities in the country. In the capital, there are 12 hospitals and 100 health centers belonging to the public sector and about 25 private hospitals. Also, in the city, there are more than 17,000 different types of healthcare professionals, of which 47% are nurses, and 14% are physicians (Deressa et al., 2021). The country’s largest COVID-19 treatment centers are located in the capital.

### Survey population and sampling

This study was conducted among practicing doctors and nurses in Addis Ababa. The inclusion criteria were direct engagement in clinical activities such as diagnosing, treating, or providing care to COVID-19 patients. The sampling frame was constructed by obtaining lists from the Ethiopian Medical and Nursing associations. A simple random sampling technique was used to select study participants from the list. In Time 1, 300 healthcare providers and in Time 2, 277 healthcare providers were surveyed. The study was designed to have the same participants in Time 1 and Time 2 however, we could only trace 73.3% of the participants in Time 2. To compensate for the lost to follow randomly selected additional participants were included in Time 2.

### Data collection

Data for both time points were collected using Computer-Assisted Telephone Interviewing (CATI) to comply with the COVID-19 protocol. The questionnaire was developed in English and then translated into Amharic. The questionnaire was adopted from the WHO questionnaire designed to assess COVID-19 related perceptions and standardized and validated tools was used to assess depression (Kroenke, Spitzer & Williams, 2001a; World Health Organization. Regional Office for Europe, 2020). The questionnaire was pretested among twenty-eight healthcare providers (5% in both surveys) who were not included in the survey to check the feasibility, validity, and interpretability of answers to the respective questions. Necessary edits were made to the tool based on the pretest results. Data were collected by trained interviewers using the local language, Amharic. The data collectors were trained for two days on the survey objectives, content of the questionnaire and research ethics. Both surveys were collected by the same data collectors. Pre-programmed tablets were used to complete the interview, and all interviews were recorded based on participants’ consent.

Depression in this study was measured using the Patient Health Questionnaire 9 (PHQ-9) tool, which is a standardized and validated tool in Ethiopia (Kroenke, Spitzer & Williams, 2001b). The PHQ-9 consists of 9 items based on the nine DSM-5 (Diagnostic and Statistical Manual of Mental Disorders) criteria for major depressive disorder (Association, 2013). Each item was scored on a four-point Likert scale, 0/not at all to 3/nearly every day. The scores range from 0 to 27, with higher scores reflecting greater depression severity. Depression symptom scores had a cutoff point of 5, 10, 15, and 20 to represent mild, moderate, moderately severe, and severe depression, respectively and a score less than 5 indicated no depression. The cut-off point of 10 was used to define the presence of depressive symptoms in this study (Gelaye et al., 2013a).

### Ethical considerations

A verbal informed consent was obtained from all participants after explaining the study’s purpose and nature. Data collectors were trained on how to maintain confidentiality and securely store data. The surveys were approved by the Institutional Ethical Review Board of the Addis Continental Institute of Public Health with reference number ACIPH/IRB/002/2020 (Time 1) and ACIPH/IRB/005/2021 (Time 2) and from Haramaya University SPH/01/180135/2021.

### Statistical analysis

A composite score was created for depression by adding up the scores of the nine PHQ-9 items. The scores ranged from 0 to 27, with a cutoff value of 10 representing an accuracy of 86% based on previous literature (Gelaye et al., 2013b). The composite score was then recorded as “0” for no depression and “1” for depression. The internal consistency of the items was good, with a Cronbach’s alpha of 0.84. All continuous variables were analyzed in descriptive terms of mean and standard deviation. Frequency and percentage were used to describe categorical variables. The association between two categorical variables was determined using Chi square test while independent t test was used for continuous variables. The prevalence estimates with 95% CIs were calculated for the outcome variable. Bivariate and multivariable logistic regression analysis was performed to identify potential factors that were associated with depression separately for Time 1 and 2. The analyses were performed using Stata version 14. The level of significance was set at *p*-value <0.05.

## Result

### Background characteristic

A total of 577 healthcare workers completed the follow-up survey, 300 in Time 1 and 277 in Time 2. The mean age of the participants was 34 ± 9.89, and more than half were included in the younger age group, which is similar across the two-time points. Female participants constituted a higher proportion, and compared to Time 1 (52.7%), there was an increase in the proportion of female participants in Time 2 (59.2%). However no significant difference was observed. The majority of the study participants, 80%, are primarily working in government health facilities and 59.8% are nurses who showed close to a 5% decrease between the two-time points (62% in Time 1 to 57.4% in Time 2) however the difference was not statistically significant.

### COVID-19 and risk perception

A majority, 86.3%, of the participants expressed concern about the spread of COVID-19, which was significantly increased at Time 2 (76% in Time 1 *vs.* 97.5% in Time 2; *p*-value <0.001). A high proportion of the study participants reported an increased perceived risk of exposure to COVID-19; risk perception was greater in Time 2 (80.7% in Time 1 *vs* 86.6% in Time 2; *p*-value 0.02). A positive COVID-19 test results; overall, 16.5% of study participants received a positive COVID-19 test result (9.0% in Time 1 *vs* 24.5% in Time 2; *p*-value <0.001). Table 1 presents detailed characteristics.

### Prevalence of depression

**Figure 1:**
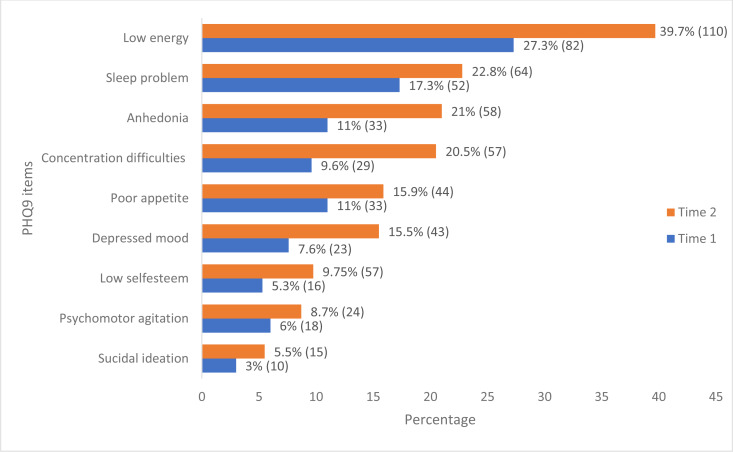
Distributional patterns of PHQ-9 item responses among healthcare professionals in Addis Ababa, Ethiopia. Each data point indicates the percentage (frequency) of reported PHQ9 symptoms in both Time 1 and Time 2.

The prevalence of depression among the study participants was 2.3% (95% CI [1.1–4.8]) at Time 1, and 6.5% (4.1–10.1) at Time 2. The prevalence has increased by nearly three-fold (2.83) between the two-time points (*p*-value 0.01). In both Time 1 and Time 2, depression was more prevalent among the younger age group (21 to 30). In Time 2, 15 (80%) of the depression was reported by female healthcare providers (Table 2). The most frequently reported symptoms in both surveys based on the PHQ-9 item were having poor energy, difficulty sleeping, anhedonia, or having little interest or enjoyment from doing things, difficulty of concentrating and change in appetite. The lowest reported item was suicidal thoughts (Fig. 1). Of the study participants, 94 (16.3%) reported various degree of depression; mild depression (11.96%), reported moderate depression (2.43%), and moderately severe or severe depression (1.91%).

**Table 1:** Background information of the participants (n=577) in Addis Ababa, Ethiopia. Data are presented as *n* (%).

**Characteristics**	**Response**	**Time 1**	**Time 2**	***p*** **-Value**
		**N=300**	**N=277**	
Individual characteristics				
Age group	21–30	165 (55)	145 (52.4)	0.2335
	31–40	72 (24)	83 (30)	
	41 +	63 (21)	49 (17.6)	
Sex	Female	158 (52.7)	164 (59.2)	0.1141
	Male	142 (47.3)	113 (40.8)	
Concerned about the spread of COVID-19	No	72 (24)	7 (2.5)	<0.001
	Yes	228 (76)	270 (97.5)	
Perceived level of risk of exposure to COVID-19	No	5 (1.7)	1 (0.4)	0.032
	Low risk	53 (17.6)	36 (13)	
	High risk	158 (52.7)	136 (49.1)	
	Very high risk	84 (28)	104 (37.6)	
Received a positive COVID-19 test result	No	273 (91)	209 (75.5)	<0.001
	Yes	27 (9)	68 (24.5)	
Profession	Physician	114 (38)	118 (42.6)	0.2612
	Nurse	186 (62)	159 (57.4)	
Primary workplace	Government Health Facility	239 (79.7)	226 (81.6)	0.5598
	Private Health Facility	61 (20.3)	51 (18.4)	
Received formal training on COVID-19	Not received training	218 (72.7)	202 (72.9)	0.9446
	Received training	82 (27.3)	75 (27.1)	
Availability of COVID-19 related workplace policy or guideline	Not available	118 (39.3)	69 (24.9)	0.0002
	Available	182 (60.7)	208 (75.1)	

**Notes.**

The numbers in the table show the frequency and percentage, *n* (%).

**Table 2:** Depression across participants demographic characteristics in Addis Ababa, Ethiopia.

**Variables**	**Depression**					
	**Time 1**			**Time 2**		
	**No**	**Yes**	***P*** **value**	**No**	**Yes**	***P*** **value**
Age group						
21–30	158 (95.7)	7 (4.2)	0.05	135 (93.1)	10 (6.9)	0.7
31–40	72 (100)	0		79 (95.2)	4 (4.8)	
41 +	63 (19.6)	0		45 (91.8)	4 (8.2)	
Sex						
Female	155 (98.1)	3 (1.9)	0.7	149 (90.9)	15 (9.1)	0.04
Male	138 (97.2)	4 (2.8)		110 (97.4)	3 (2.7)	
Profession						
Doctors	109 (95.6)	5 (4.4)	0.1	109 (92.4)	9 (7.6)	0.4
Nurses	184 (98.9)	2 (1.1)		150 (94.3)	9 (5.7)	
Primary workplace				
Government Health Facility	232 (97.1)	7 (2.9)	0.3	210 (92.9)	16 (7.1)	0.5
Private Health Facility	61 (100)	0		49 (96.1)	2 (3.9)	
Received formal training on COVID-19					
No	215 (98.6)	3 (1.8)	0.09	190 (94.1)	12 (5.9)	0.5
Yes	78 (9.1)	4 (4.9)		69 (92)	6 (8)	
Availability of COVID-19 related Policy or guidance at the workplace					
No	114 (96.6)	4 (3.4)	0.4	61 (88.4)	8 (11.6)	0.06
Yes	179 (98.4)	3 (1.6)		198 (95.2)	10 (4.8)	
Received a positive COVID-19 test result					
No	269 (98.5)	4 (1.5)	0.01	196 (93.8)	13 (6.2)	0.8
Yes	24 (88.9)	3 (11.1)		63 (92.7)	5 (7.3)	
Concerned about the spread of COVID-19						
No	71 (98.6)	1 (1.4)	1.00	6 (85.7)	1 (14.3)	0.3
Yes	222 (97.4)	6 (2.6)		253 (93.7)	17 (6.3)	
Perceived level of risk of exposure to COVID-19						
Low risk	208 (98.6)	3 (1.4)	0.1	158 (91.9)	14 (8.1)	0.2
High risk	80 (95.2)	4 (4.8)		100 (96.2)	4 (3.8)		

**Notes.**

The numbers in the table show the frequency and percentage, *n* (%).

### Factors associated with depression

The multivariable logistic regression model fitted separately for the two time points revealed that having a positive COVID-19 test result (AOR 7.21 95% CI [1.32–39.39]) was significantly associated with depression in Time 1(Table 3A). Being female (AOR 3.96 95% CI [1.08–14.51]) and the lack of COVID-19 related policies or guidelines at the workplace (AOR 3.22 95% CI [1.11–9.35]) were significantly associated with depression in Time 2 (Table 3B).

**Table 3:** Bivariable and multivariable analyses of factors associated with depression among healthcare professionals in Addis Ababa, Ethiopia. (A) Bivariable *p*-value = 0.007 and *p*-value = 0.02 in Multivariable (Received a positive COVID-19 test result) (B) Bivariable *p*-value = 0.04 for Sex. Multivariable *p*-value = 0.03 for both Sex and Availability of COVID-19 related guideline.

A		
**Variables** **(Time 1, *n* = 300)**	**COR (95% CI)**	**AOR (95% CI)**
Sex		
Male	1.00	1.00
Female	0.66 (0.15–3.04)	1.12 (0.22–5.55)
Profession		
Doctors	1.00	1.00
Nurses	0.23 (0.04–1.24)	0.26 (0.04–1.48)
Received formal training on COVID-19		
No	0.27 (0.06–1.24)	0.20 (0.04–1.15)
Yes	1.00	1.00
Availability of COVID-19 related Policy or guidance at the workplace		
No	2.09 (0.46–9.52)	0.20 (0.04–1.15)
Yes	1.00	1.00
Received a positive COVID-19 test result		
No	1.00	1.00
Yes	8.4 (1.77–39.76)*	7.21 (1.32–39.39)*

B		
**Variables** **(Time 2, *n* = 277)**	**COR (95% CI)**	**AOR (95% CI)**
Age group		
21–30	1.00	1.00
31–40	0.68 (0.21–2.25)	0.78 (0.22–2.71)
41 +	1.2 (0.36–4.01)	1.82 (0.50–6.61)
Sex		
Male	1.00	1.00
Female	3.69 (1.04–13.06)*	3.96 (1.08–14.51)*
Profession		
Doctors	1.00	1.00
Nurses	0.73 (0.28–1.89)	0.63 (0.22–1.81)
Primary workplace		
Government Health Facility	1.00	1.00
Private Health Facility	0.54 (0.12–2.41)	0.42 (0.09–1.98)
Received formal training on COVID-19		
No	0.72 (0.26–2.01)	0.52 (0.17–1.64)
Yes	1.00	1.00
Availability of COVID-19 related Policy or guidance at the workplace		
No	2.59 (0.98–6.87)	3.22 (1.11–9.35)*
Yes	1.00	1.00
Received a positive COVID-19 test result		
No	1.00	1.00
Yes	1.19 (0.41–3.48)	0.92 (0.29–2.84)

**Notes.**

The asterisks indicate the level of significance.

## Discussion

In this study, the prevalence of depression among healthcare professionals in Ethiopia was examined at two-time points in a one-year interval. The prevalence of depression among healthcare workers tripled in a year (between Time 1 and Time 2). Depression was significantly associated with having positive COVID-19 test result in Time 1. While in Time 2, being female and absence of COVID-19 related guidelines in the workplace; the odds of having depression was three times greater among female HCPs and those working in facilities where there were no COVID-19 policies and guidelines.

The global estimates of depression among healthcare professionals ranged from 22.8%–51.0% (Olaya et al., 2021; Pappa et al., 2020; Rezaei et al., 2022). Among the WHO regions, the greatest prevalence of depression among HCPs was reported in the African region ranging from 12.5% to 82% (Debes et al., 2021; Moitra et al., 2021; Olashore et al., 2021; Rezaei et al., 2022). However, compared to other studies undertaken in Africa and other LMICs, the overall prevalence of depression reported in this study was low (Hayat et al., 2021; Wilson et al., 2020). Studies carried out in Ethiopia also reported a higher prevalence which varied from 20% to 60% (Ayalew et al., 2021; Jemal et al., 2021; Mulatu et al., 2021; Wayessa et al., 2021). The differences in the prevalence of depression observed in these studies may result from the use of various screening tools, survey time, rating scales, or cutoff scores to identify clinically significant levels of depression (Belete & Anbesaw, 2022). Studies that used lower cutoff levels reported a higher proportion of depression, which could have magnified the reported prevalence. In addition, study context and techniques that place a greater emphasis on subgroups of HCPs could have increased the depression prevalence reported by other studies (Bitew et al., 2022; Lai et al., 2020; Peccoralo et al., 2022; Th’ng et al., 2021; Zhou et al., 2021). Different evidences showed that the availability of the COVID-19 vaccine has led to improvements in mental health status (Al-Obaidy, Attash & Al-Qazaz, 2022; Koltai et al., 2022). In Ethiopia, HCPs were privileged to get the COVID-19 vaccines before the general population during the data collection points, and the perceived protection as a result of the vaccination could have resulted in lower depression.

The data collection technique used in this study, that is administering the PHQ-9 screening tool using telephone interviews could have contributed to the observed low prevalence of depression (Pinto-Meza et al., 2005). Higher levels of risk perception observed in this study may also have contributed to the lower level of depression, which is in accordance with earlier studies (Almeida et al., 2021; Assefa et al., 2021). The risk of infecting family members is one of the major stressors among HCPs which creates fear of transmitting the virus to their family members, especially those working in COVID-19 treatment centers (Asa et al., 2022; De Kock et al., 2021; Mulatu et al., 2021). In order to address this, the government of Ethiopia initiated early support for healthcare providers through the arrangement of places (such as hotels or camps) for those who have direct contact with COVID-19 patients, along with adequate compensation. This support continued until they completed their duties and isolation times before joining their families. This strengthened support by the government could have contributed to the low prevalence in this study (Cai et al., 2020; Federal Ministry of Health of Ethiopia, 2020).

The increased prevalence of depression observed in time two is consistent with observations in other countries (Elliott et al., 2021; Jemal et al., 2021; Zhou et al., 2021). Infectious disease epidemics that run a longer course are significantly associated with long-term mental health issues, including depression among healthcare providers (Lee et al., 2007; Zara et al., 2021). The mental health impact of COVID-19 is greatly dependent on the severity and outcome of the pandemic (Hajek et al., 2022). COVID-19 variants with higher transmission rates and virulence in subsequent waves could be the reason for the increased prevalence of depression in our second survey. The number of COVID-19 related deaths reached a record high, and also the number of confirmed COVID-19 cases greatly increased in Ethiopia during the second survey time. Having poor energy was primarily reported symptom by the study participants, followed by difficulty sleeping, which could have augmented the burnout levels of healthcare providers. Burnouts due to excessive workloads compounded by a shortage of healthcare personnel and other resources may have contributed to the relatively increased prevalence of depression (Debes et al., 2021; Jaber et al., 2022; Zhou et al., 2021).

Our finding that showed a positive association between positive COVID-19 test and depression was consistent with previous studies (Almalki et al., 2021; Magnavita, Tripepi & Prinzio, 2020). During the first survey, fear of the pandemic was very high in Ethiopia and that could have contributed to the higher level of depression among HCPs. Additionally, isolations, hospitalizations, fear of transmitting the virus to family/loved ones, and fatigue could have all contributed to increased depression. Our study related to gender was also consistent with previous studies that showed an increased occurrence of depression (Jemal et al., 2021; Wilson et al., 2020) which was attributed to a larger female proportion of hospital staff, hormonal fluctuations, and higher family responsibilities that women carry especially in low-income countries (Elliott et al., 2021; Mulatu et al., 2021).

Having comprehensive COVID-19 guidelines at the workplace is among the protective factors for depression (Maraqa, Nazzal & Zink, 2020; Olashore et al., 2021). Unavailability of COVID-19 guidelines, insufficient training, and lack of resources were reported as the primary occupational risk factors for pandemic-related mental health issues among HCPs (Wang et al., 2020; Olashore et al., 2021). Consistently, our study revealed that the odds of developing depression were three times higher among study participants who reported lack of COVID-19 related guidelines (Bitew et al., 2022; Dawood, Tomita & Ramlall, 2022; Olashore et al., 2021). Trained study participants had a greater risk of having depression though our finding showed no statistically significant difference in developing depression between study participants who received COVID-19 training and those who did not. Previous studies have revealed that the quality and shorter duration of COVID-19 related training is a reason for high depression among trained HCPs (Beneria et al., 2020; Bitew et al., 2022; Wang et al., 2020).

The limitations of this study include the selection of study participants who were members of their respective professional associations and sample size was limited due to time constraints. Thus, the findings may not be generalizable to the wider category of healthcare professionals in the study area and certainly not generalizable to other locations in the country, especially rural areas. The information collected was self-reported and could have been exposed to social desirability bias. This study did not evaluate other variables that may have an impact on depression, such as burnout, resilience, economic position, or sleep problems. Despite these limitations, our study gives valuable insights into healthcare workers’ levels of depression in a year’s time.

## Conclusion

Our study revealed a three-fold increase in the prevalence of depression in a one-year period. While testing positive for COVID-19 test was significantly associated at Time 1, at Time 2 being female and lack of COVID-19 guidelines were significant factors. Thus, panic appeared to have more mental health impact at the beginning and the lack of disease-specific guidelines appeared to have affected health workers more in the long-run. Having poor energy, sleep problems, and having little interest from doing things are the most frequently reported symptoms. To mitigate the mental health impact of the COVID-19 pandemic, appropriate and timely workplace interventions are essential.

##  Supplemental Information

10.7717/peerj.15053/supp-1 Data S1 Depression among HCPs

## Additional Information and Declarations

### Data Availability

The following information was supplied regarding data availability: The raw data is available in the Supplemental File.
